# Dissecting Long-Term Adjustments of Photoprotective and Photo-Oxidative Stress Acclimation Occurring in Dynamic Light Environments

**DOI:** 10.3389/fpls.2016.01690

**Published:** 2016-11-09

**Authors:** Shizue Matsubara, Trang Schneider, Veronica G. Maurino

**Affiliations:** ^1^IBG-2: Plant Sciences, Institute of Bio- and Geosciences, Forschungszentrum JülichJülich, Germany; ^2^iGRAD-Plant, Heinrich-Heine-UniversitätDüsseldorf, Germany; ^3^Institute of Developmental and Molecular Biology of Plants, Plant Molecular Physiology and Biotechnology Group, Heinrich-Heine-Universität and Cluster of Excellence on Plant SciencesDüsseldorf, Germany

**Keywords:** acclimation, fluctuating light, photoprotection, reactive oxygen species, retrograde signaling

## Abstract

Changes in light intensity directly affect the performance of the photosynthetic apparatus. Light energy absorbed in excess of cells’ needs leads to production of reactive oxygen species and photo-oxidative damage. Excess light in both constant and dynamic environments induces photoprotective acclimation in plants. Distinct sets of signals and regulatory mechanisms are involved in acclimatory adjustment of photoprotection and photosynthesis under constant and dynamic (fluctuating) light conditions. We are still far away from drawing a comprehensive picture of acclimatory signal transduction pathways, particularly in dynamic environments. In this perspective article, we propose the use of *Arabidopsis* plants that produce H_2_O_2_ in chloroplasts (GO plants) under atmospheric CO_2_ levels as a tool to study the mechanisms of long-term acclimation to photo-oxidative stress. In our opinion there are new avenues to future investigations on acclimatory adjustments and signal transduction occurring in plants under dynamic light environments.

## Acclimation to Photo-Oxidative Stress is Induced By Fluctuating Light

Rapid climate changes and transformation of landscapes by extensive agricultural practices impose environmental perturbations. Plants in the affected areas respond to the perturbations through acclimation (within generation) or adaptation (over generations). Light intensity can vary rapidly by a few orders of magnitude as clouds travel in the sky or wind moves outer canopy leaves and taller plants. Especially, wind can briefly expose inner canopy leaves and understory plants to intense sunlight. Upon large and abrupt increase in light intensity, photosynthetic light energy utilization is limited biochemically. This is attributed to low availability of the Calvin-Benson cycle intermediates, low activation state of Ribulose-1,5-bisphosphate carboxylase/oxygenase (RubisCO), and also low stomatal conductance measured in leaves under low light (LL) conditions (Kirschbaum and Pearcy, 1988; Pearcy, 1990).

**FIGURE 1:**
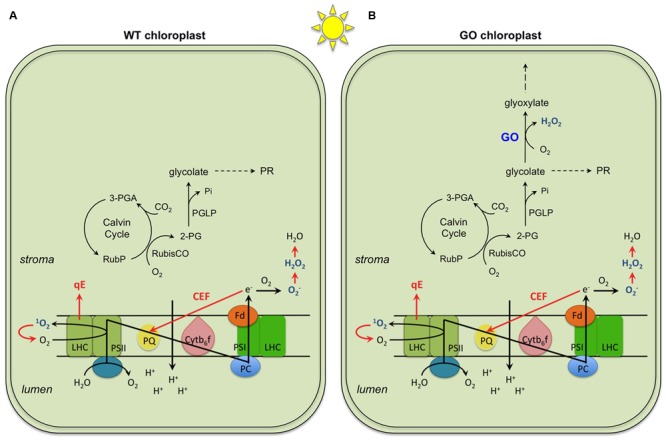
**General mechanisms of photoprotection (highlighted in red) occurring in chloroplasts of wild type (WT)** **(A)** and the GO plants **(B)**. Three mechanisms represented are (i) qE, the thermal energy dissipation of excess absorbed light at the light harvesting complex (LHC) of Photosystem II (PSII); (ii) CEF, the cyclic electron flow around Photosystem I (PSI), and (iii) enzymatic and non-enzymatic ROS scavenging through production of O_2_, H_2_O_2_ and H_2_O. These mechanisms operate both in FL and CL; the only difference demonstrated so far is a more dramatic effect of CEF in FL than in CL. Shown in or around the thylakoid membrane are the components of photosynthetic electron transport chain: plastoquinone (PQ), cytochrome b_6_f complex (Cytb_6_f), plastocyanin (PC), and ferredoxin (Fd). In addition, the activities of RubisCO as carboxylase and oxygenase in light and under ambient CO_2_ and O_2_ conditions are shown. In the WT, the 2-phosphoglycolate (2-PG) produced initiates the photorespiratory cycle (PR; not shown in detail in the figure; for details see (Maurino and Peterhansel, 2010)). In the GO plants, 2-PG is delivered to the PR and the glycolate produced in the chloroplasts is also used by glycolate oxidase (GO) generating H_2_O_2_. 3-PGA, 3-phosphoglycerate; PGLP: 2-phosphoglycolate phosphatase; RubP, ribulose 1-5,bisphosphate.

When light energy is absorbed by photosynthetic pigments in excess of cells’ needs for reducing equivalents and chemical energy (excess light, EL), it can lead to production of reactive oxygen species (ROS) and photo-oxidative damage in oxygenic photosynthetic organisms. A range of mechanisms have evolved to reduce uncontrolled production of ROS and to protect the photosynthetic apparatus against their detrimental effects (Foyer and Noctor, 2000; Endo and Asada, 2008; Li et al., 2009). These include thermal energy dissipation which is rapidly induced in light-harvesting antenna complexes by a proton concentration gradient (ΔpH) across the thylakoid membrane (termed qE), alternative sinks for excess electrons (e.g., water-water cycle and cyclic electron flows around photosystem I, CEF) which contribute to ΔpH formation especially when liner electron transport rate (ETR) is low, and enzymatic and non-enzymatic antioxidative systems which detoxify ROS (**Figure 1A**).

When EL conditions persist, plants are able to augment their photoprotective capacities via long-term acclimation. In general, multiple mechanisms of photoprotective acclimation (**Figure 1A**) operate in plants under constant as well as dynamic EL environments. In particular, fluctuating light (FL) with short periods of EL (i.e., dynamic EL) induces, primarily or initially, long-term acclimatory changes that are characterized by improved protection against photo-oxidative stress and reduced carbon gain (Alter et al., 2012). For instance, LL-grown *Arabidopsis* plants upregulate photoprotection in highly dynamic EL conditions without developing symptoms of severe photo-oxidative injuries, such as strong photoinhibition or bleaching (Alter et al., 2012). Similar photoprotective responses are also seen during acclimation to high light (HL, i.e., constant EL), although in HL they are often accompanied by enhancement of photosynthesis and thus increased carbon gain (Leakey et al., 2002; Alter et al., 2012). There seems to be an inverse relationship between the maximum photosynthetic capacity, which is developed through photosynthetic acclimation, and the frequency of LL-HL transitions (Retkute et al., 2015). Selective upregulation of photoprotection, but not photosynthesis, in highly dynamic FL suggests that distinct sets of signals and regulatory mechanisms are involved in acclimatory adjustment of photoprotection and photosynthesis, and that signal molecules, which trigger photo-oxidative stress acclimation, are produced in leaves under the FL conditions. In this article we use the term “FL” to refer to highly dynamic EL conditions, while we are aware that FL may not always cause EL and photo-oxidative stress, depending on the amplitude and frequency of light fluctuations (Yin and Johnson, 2000; Alter et al., 2012; Retkute et al., 2015).

## Chloroplast Retrograde Signaling is Involved in Photo-Oxidative Stress Acclimation

Today it is widely recognized that ROS not only can damage cellular components but also act as signals to induce abiotic and biotic stress responses (Foyer and Noctor, 2000; Apel and Hirt, 2004; Mittler et al., 2011; Karpinski et al., 2013; Dietz et al., 2016). Multiple ROS can be generated in chloroplasts under photo-oxidative stress, such as singlet oxygen (^1^O_2_), superoxide anion radical ($O_{2}^{•–}$), hydroxyl radical (OH), and hydrogen peroxide (H_2_O_2_) (Asada, 1999; Foyer and Noctor, 2000). Among these, H_2_O_2_ alone would be able to diffuse into the cytosol because high reactivity and charge of the other species prevent them from diffusing a long distance across chloroplast envelopes. Intracellular H_2_O_2_ signaling engages both compartment-specific and non-specific pathways. In the case of retrograde signaling, chloroplastic H_2_O_2_ produced by ectopic overexpression of glycolate oxidase (GO) in chloroplasts (Fahnenstich et al., 2008) (**Figure 1B**) induces transcriptional changes in the nucleus, which partly, but not fully, overlap with the responses to peroxisomal H_2_O_2_ (Balazadeh et al., 2012; Sewelam et al., 2014).

Though ^1^O_2_ may not move far, it can give rise to secondary messengers by reacting with nearby molecules such as β-carotene (Ramel et al., 2013). Oxidation of β-carotene produces β-cyclocitral, a reactive electrophile species that can modify transcription of ^1^O_2_-responsive genes in the nucleus (Havaux, 2014). Whilst some β-carotenes are continuously oxidized and degraded in thylakoids during illumination with or without EL (Beisel et al., 2010), elevated production of ^1^O_2_ and thus β-cyclocitral under photo-oxidative stress may trigger acclimatory responses that are distinct from ^1^O_2_-induced cell death (op den Camp et al., 2003).

The number as well as the variety of agents implicated in chloroplast retrograde signaling have been increasing in the last years. For example, the redox state of the plastoquinone pool, different metabolites (e.g., tetrapyrroles, phosphoadenosine phosphate, and methylerythritol cyclodiphosphate) and hormones (abscisic acid, salicylic acid, and jasmonic acid) are regarded as such signaling agents to trigger long-term acclimatory adjustments (Dietz and Pfannschmidt, 2011; Sun et al., 2011; Estavillo et al., 2012; Xiao et al., 2012; Barajas-Lopez Jde et al., 2013; Karpinski et al., 2013; Dietz, 2015; Laloi and Havaux, 2015). To reconstruct signaling networks from individual components and pathways is a major challenge in understanding time-dependent regulation and interaction of stress response networks in plants (Dietz, 2015). Acclimation to photo-oxidative stress has been studied extensively in the context of HL or constant EL acclimation, in which plants manifest parallel enhancement of photoprotection and photosynthesis alongside other responses related to temperature and/or water stress. Highly dynamic FL, which predominantly elicits photoprotective responses (Alter et al., 2012), offers a complementary approach to investigate signals and pathways that are primarily engaged in photo-oxidative stress acclimation.

## *Arabidopsis* Plants that Produce H_2_O_2_ in Chloroplasts are a Model to Study Photo-Oxidative Stress Acclimation and Signaling

**FIGURE 2:**
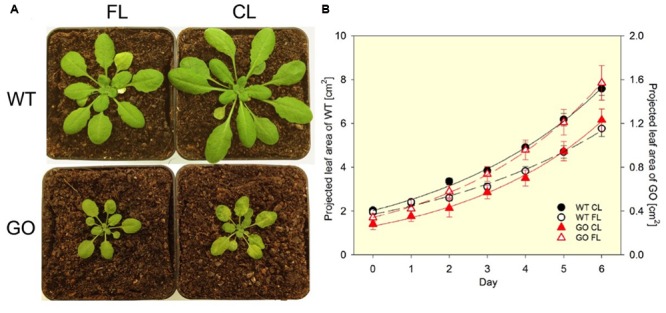
**Phenotypes of wild-type (WT) and GO plants grown under fluctuating light (FL) and constant light (CL) conditions in ambient CO_2_.(A)** Under FL (switching between ∼50 μmol photons m^-2^ s^-1^ for 280 s and ∼1000 μmol photons m^-2^ s^-1^ for 20 s) the GO plants have normal green leaves, while under CL (∼75 μmol photons m^-2^ s^-1^) they develop pale-green patches due to the chloroplastic production of H_2_O_2_. The pictures were taken after 8-days exposure to FL or CL. **(B)** The FL condition reduces leaf growth of the WT plants (left y-axis). The GO plants (right y-axis) are much smaller than the WT and the exposure to FL does not impair growth in the GO plants compared to the CL condition. Projected leaf area is the leaf area that is visible in a 2D top-view image. Solid and broken lines show exponential regression for the CL and FL data sets, respectively. The *R*^2^ values of the fitting are: 0.996 (WT CL), 0.991 (WT FL), 0.997 (GO CL), and 0.999 (GO FL). Relative growth rates (% d^-1^) obtained by the regression are: 22.1 ± 0.6 (WT CL), 18.7 ± 0.8 (WT FL), 25.7 ± 0.7 (GO CL), and 25.4 ± 0.3 (GO FL). The difference in relative growth rate between CL and FL is significant for the WT (*P* = 0.002). *n* = 16 (WT) and 9 (GO).

*Arabidopsis* plants, in which GO is targeted to chloroplasts, generate H_2_O_2_ in chloroplasts under ambient CO_2_ concentrations (photorespiratory conditions) (Fahnenstich et al., 2008; Strand et al., 2015). Because the GO reaction depends on the substrate provided by the oxygenase activity of RubisCO in the light (**Figure 1B**), the level of GO-dependent H_2_O_2_ production can be controlled by changing the growth conditions. When growing in LL (75 μmol photons m^-2^ s^-1^) and ambient CO_2_ concentration (380 ppm), the GO plants are smaller than the wild-type (WT) plants and present patchy pale-green leaf lamina (**Figure 2A**; constant light, CL) as a result of H_2_O_2_ production in chloroplasts and overload of the antioxidant machinery (Fahnenstich et al., 2008). Under HL the GO plants develop severe oxidative lesions and ultimately bleach, whereas combinations of very LL (30 μmol photons m^-2^ s^-1^) and ambient CO_2_ or LL and high CO_2_ (4,000 ppm) allow them to grow like WT (Fahnenstich et al., 2008; Balazadeh et al., 2012; Sewelam et al., 2014). Also, they become as big and green as WT and recover the WT level of photosynthetic ETR in LL when transferred from ambient to high CO_2_ conditions (Fahnenstich et al., 2008). These features make the GO plants a unique, well-established model to study the action of H_2_O_2_ in chloroplast retrograde signaling (Balazadeh et al., 2012; Sewelam et al., 2014).

Interestingly, FL conditions applied to the GO plants growing in ambient CO_2_ —in spite of (or maybe because of) the effect of FL to impose photo-oxidative stress— provoke disappearance of the characteristic patchy pale-green phenotype of these plants (**Figure 2A**). This reversion to normal green leaf lamina under FL allows the GO plants to maintain similar relative growth rates in CL and FL, whereas WT exhibits growth reduction in FL (**Figure 2B**). The FL-induced recovery of leaf color in the GO plants is most probably due to upregulation of H_2_O_2_ scavenging in the chloroplast, as the patchy pale-green phenotype is a consequence of H_2_O_2_ accumulation (Fahnenstich et al., 2008). This assumption, i.e., FL-induced acclimatory enhancement of ROS (H_2_O_2_) scavenging systems in the GO plants, is supported by the observations made in carotenogenic mutants of *Arabidopsis*; despite having reduced capacities for qE and carotenoid-dependent ROS (mainly ^1^O_2_) scavenging, these mutants do not suffer from chronic photo-oxidative damage under FL conditions because they can upregulate other photoprotective and ROS scavenging mechanisms by long-term acclimation (Caliandro et al., 2013). The increased activity of superoxide dismutase (SOD) found in leaves of WT following FL acclimation (Alter et al., 2012) also points to an increased detoxification capacity for H_2_O_2_ which arises from disproportionation of $O_{2}^{•–}$ catalyzed by SOD.

Thus, we hypothesize that long-term acclimatory upregulation of H_2_O_2_ scavenging occurs in chloroplasts under FL conditions. H_2_O_2_ reduction in chloroplasts could proceed mainly via peroxidase systems, including glutathione peroxidase (Gpx) and peroxiredoxin (Prx) in the stroma, as well as thylakoid-bound and stromal ascorbate peroxidase (tAPX, sAPX) coupled to monodehydroascorbate reductase (MDHAR) and also dehydroascorbate reductase (DHAR) (Foyer and Noctor, 2011). Gpx and Prx use thiol-based peroxide-detoxification mechanisms that are maintained by glutathione and glutathione reductase (GR) (Dietz and Pfannschmidt, 2011). Regeneration of ascorbate by DHAR is also dependent on glutathione and GR, while MDHAR uses NAD(P)H to regenerate ascorbate. In addition to the removal of H_2_O_2_, other photoprotective mechanisms such as CEF and carotenoid-dependent reactions could also contribute to the rescuing of the GO phenotype in FL by keeping the level of ROS production under control (**Figure 1**). Indeed, CEF is activated by chloroplastic H_2_O_2_ produced in HL (Strand et al., 2015) and carotenoid contents (especially xanthophyll-cycle pigments) increase in leaves exposed to FL (Alter et al., 2012; Caliandro et al., 2013).

## Future Directions

So far it is not known whether, and if yes, which H_2_O_2_ scavenging pathways are upregulated in chloroplasts during FL acclimation. This could be studied by analyzing antioxidant defense systems in the GO plants during acclimation to CL and FL conditions. An important question that can then be tackled is the long-term acclimation of H_2_O_2_ scavenging systems. Signal agents, which are generated in FL conditions and lead to acclimatory enhancement of H_2_O_2_ scavenging, must be different from the signals induced by chloroplastic H_2_O_2_ produced in the GO plants under CL and ambient CO_2_ levels. Close inspections of the GO plants during FL acclimation at different response levels —from gene expression, protein and metabolite accumulation to physiological phenotype— could shed light on components and signal agents involved in upregulation of H_2_O_2_ scavenging under photo-oxidative stress. Once candidate molecules are identified, the unique feature of the GO plants, which visualizes acclimatory changes in H_2_O_2_ metabolism under FL, can be exploited again as the genetic background to assess the efficacy of those molecules in upregulating H_2_O_2_ detoxification. The nature of chloroplast retrograde signaling in FL (dynamic EL), as compared with that in HL (constant EL), inspires further experiments and investigations.

## Author Contributions

SM and VM contributed equally to writing the manuscript. TS performed the growth analysis shown in **Figure 2**.

## References

[B1] Alter P. Dreissen A. Luo F. L. Matsubara S. (2012). Acclimatory responses of *Arabidopsis* to fluctuating light environment: comparison of different sunfleck regimes and accessions. *Photosynth. Res.* 113 221–237. 10.1007/s11120-012-9757-2 22729524 PMC3430843

[B2] Apel K. Hirt H. (2004). Reactive oxygen species: metabolism, oxidative stress, and signal transduction. *Annu. Rev. Plant Biol.* 55 373–399. 10.1146/annurev.arplant.55.031903.141701 15377225

[B3] Asada K. (1999). The water-water cycle in chloroplasts: scavenging of active oxygens and dissipation of excess photons. *Annu. Rev. Plant Physiol. Plant Mol. Biol.* 50 601–639. 10.1146/annurev.arplant.50.1.601 15012221

[B4] Balazadeh S. Jaspert N. Arif M. Mueller-Roeber B. Maurino V. G. (2012). Expression of ROS-responsive genes and transcription factors after metabolic formation of H_2_O_2_ in chloroplasts. *Front. Plant Sci.* 3:234 10.3389/fpls.2012.00234 PMC3485569 23125844

[B5] Barajas-Lopez Jde D. Blanco N. E. Strand A. (2013). Plastid-to-nucleus communication, signals controlling the running of the plant cell. *Biochim. Biophys. Acta* 1833 425–437. 10.1016/j.bbamcr.2012.06.020 22749883

[B6] Beisel K. G. Jahnke S. Hofmann D. Koppchen S. Schurr U. Matsubara S. (2010). Continuous turnover of carotenes and chlorophyll a in mature leaves of *Arabidopsis* revealed by 14CO2 pulse-chase labeling. *Plant Physiol.* 152 2188–2199. 10.1104/pp.109.151647 20118270 PMC2850008

[B7] Caliandro R. Nagel K. A. Kastenholz B. Bassi R. Li Z. Niyogi K. K. (2013). Effects of altered alpha- and beta-branch carotenoid biosynthesis on photoprotection and whole-plant acclimation of *Arabidopsis* to photo-oxidative stress. *Plant Cell Environ.* 36 438–453. 10.1111/j.1365-3040.2012.02586.x 22860767 PMC3640260

[B8] Dietz K. J. (2015). Efficient high light acclimation involves rapid processes at multiple mechanistic levels. *J. Exp. Bot.* 66 2401–2414. 10.1093/jxb/eru505 25573858

[B9] Dietz K. J. Pfannschmidt T. (2011). Novel regulators in photosynthetic redox control of plant metabolism and gene expression. *Plant Physiol.* 155 1477–1485. 10.1104/pp.110.170043 21205617 PMC3091116

[B10] Dietz K. J. Turkan I. Krieger-Liszkay A. (2016). Redox- and reactive oxygen species-dependent signaling into and out of the photosynthesizing chloroplast. *Plant Physiol.* 171 1541–1550. 10.1104/pp.16.00375 27255485 PMC4936569

[B11] Endo T. Asada K. (2008). “Photosystem I and photoprotection: cycling electron flow and water-water cycle,” in *Photoprotection, Photoinhibition, Gene Regulation, and Environment* eds Demmig-Adams B. Adams W. W. Wattoo A. K. (Berlin: Springer) 205–211.

[B12] Estavillo G. M. Chan K. X. Phua S. Y. Pogson B. J. (2012). Reconsidering the nature and mode of action of metabolite retrograde signals from the chloroplast. *Front. Plant Sci.* 3:300 10.3389/fpls.2012.00300 PMC3539676 23316207

[B13] Fahnenstich H. Scarpeci T. E. Valle E. M. Flugge U. I. Maurino V. G. (2008). Generation of hydrogen peroxide in chloroplasts of *Arabidopsis* overexpressing glycolate oxidase as an inducible system to study oxidative stress. *Plant Physiol.* 148 719–729. 10.1104/pp.108.126789 18685041 PMC2556821

[B14] Foyer C. H. Noctor G. (2000). Oxygen processing in photosynthesis: regulation and signalling. *New Phytol.* 146 359–388. 10.1046/j.1469-8137.2000.00667.x

[B15] Foyer C. H. Noctor G. (2011). Ascorbate and glutathione: the heart of the redox hub. *Plant Physiol.* 155 2–18. 10.1104/pp.110.167569 21205630 PMC3075780

[B16] Havaux M. (2014). Carotenoid oxidation products as stress signals in plants. *Plant J.* 79 597–606. 10.1111/tpj.12386 24267746

[B17] Karpinski S. Szechynska-Hebda M. Wituszynska W. Burdiak P. (2013). Light acclimation, retrograde signalling, cell death and immune defences in plants. *Plant Cell Environ.* 36 736–744. 10.1111/pce.12018 23046215

[B18] Kirschbaum M. U. Pearcy R. W. (1988). Gas exchange analysis of the relative importance of stomatal and biochemical factors in photosynthetic induction in *Alocasia macrorrhiza*. *Plant Physiol.* 86 782–785. 10.1104/pp.86.3.782 16665988 PMC1054570

[B19] Laloi C. Havaux M. (2015). Key players of singlet oxygen-induced cell death in plants. *Front. Plant Sci.* 6:39 10.3389/fpls.2015.00039 PMC4316694 25699067

[B20] Leakey A. D. B. Press M. C. Scholes J. D. Watling J. R. (2002). Relative enhancement of photosynthesis and growth at elevated CO2 is greater under sunflecks than uniform irradiance in a tropical rain forest tree seedling. *Plant Cell Environ.* 25 1701–1714. 10.1046/j.1365-3040.2002.00944.x

[B21] Li Z. Wakao S. Fischer B. B. Niyogi K. K. (2009). Sensing and responding to excess light. *Annu. Rev. Plant Biol.* 60 239–260. 10.1146/annurev.arplant.58.032806.103844 19575582

[B22] Maurino V. G. Peterhansel C. (2010). Photorespiration: current status and approaches for metabolic engineering. *Curr. Opin. Plant Biol.* 13 249–256. 10.1016/j.pbi.2010.01.006 20185358

[B23] Mittler R. Vanderauwera S. Suzuki N. Miller G. Tognetti V. B. Vandepoele K. (2011). ROS signaling: the new wave? *Trends Plant Sci.* 16 300–309. 10.1016/j.tplants.2011.03.007 21482172

[B24] op den Camp R. G. Przybyla D. Ochsenbein C. Laloi C. Kim C. Danon A. (2003). Rapid induction of distinct stress responses after the release of singlet oxygen in *Arabidopsis*. *Plant Cell* 15 2320–2332. 10.1105/tpc.014662 14508004 PMC197298

[B25] Pearcy R. W. (1990). Sunflecks and photosynthesis in plant canopies. *Annu. Rev. Plant Physiol. Plant Mol. Biol.* 41 421–453. 10.1146/annurev.pp.41.060190.002225

[B26] Ramel F. Mialoundama A. S. Havaux M. (2013). Nonenzymic carotenoid oxidation and photooxidative stress signalling in plants. *J. Exp. Bot.* 64 799–805. 10.1093/jxb/ers223 22915744

[B27] Retkute R. Smith-Unna S. E. Smith R. W. Burgess A. J. Jensen O. E. Johnson G. N. (2015). Exploiting heterogeneous environments: does photosynthetic acclimation optimize carbon gain in fluctuating light? *J. Exp. Bot.* 66 2437–2447. 10.1093/jxb/erv055 25788730 PMC4629418

[B28] Sewelam N. Jaspert N. Van der Kelen K. Tognetti V. B. Schmitz J. Frerigmann H. (2014). Spatial H2O2 signaling specificity: H2O2 from chloroplasts and peroxisomes modulates the plant transcriptome differentially. *Mol. Plant* 7 1191–1210. 10.1093/mp/ssu070 24908268

[B29] Strand D. D. Livingston A. K. Satoh-Cruz M. Froehlich J. E. Maurino V. G. Kramer D. M. (2015). Activation of cyclic electron flow by hydrogen peroxide in vivo. *Proc. Natl. Acad. Sci. U.S.A.* 112 5539–5544. 10.1073/pnas.1418223112 25870290 PMC4418880

[B30] Sun X. Feng P. Xu X. Guo H. Ma J. Chi W. (2011). A chloroplast envelope-bound PHD transcription factor mediates chloroplast signals to the nucleus. *Nat. Commun.* 2:477 10.1038/ncomms1486 21934661

[B31] Xiao Y. Savchenko T. Baidoo E. E. Chehab W. E. Hayden D. M. Tolstikov V. (2012). Retrograde signaling by the plastidial metabolite MEcPP regulates expression of nuclear stress-response genes. *Cell* 149 1525–1535. 10.1016/j.cell.2012.04.038 22726439

[B32] Yin Z. H. Johnson G. N. (2000). Photosynthetic acclimation of higher plants to growth in fluctuating light environments. *Photosynth. Res.* 63 97–107. 10.1023/A:1006303611365 16252168

